# Supplementary material for: Complete attenuation of Plasmodium falciparum sporozoites by atovaquone–proguanil
Source: EMBO Mol Med. 2025 Sep 29;17(11):2875–900. doi: 10.1038/s44321-025-00301-8 (PMC12602697; doi:10.1038/s44321-025-00301-8)
Supplement: Supplementary file 1 — Appendix [file 44321_2025_301_MOESM1_ESM.pdf]

Appendix Borrmann *et al.*

Table of Contents:

	Page number
Appendix Figure S1	2
Appendix Table S1	3
Appendix Table S2	4
Appendix Table S3	6
Appendix Table S4	8
Appendix Table S5	11

Appendix Figure S1

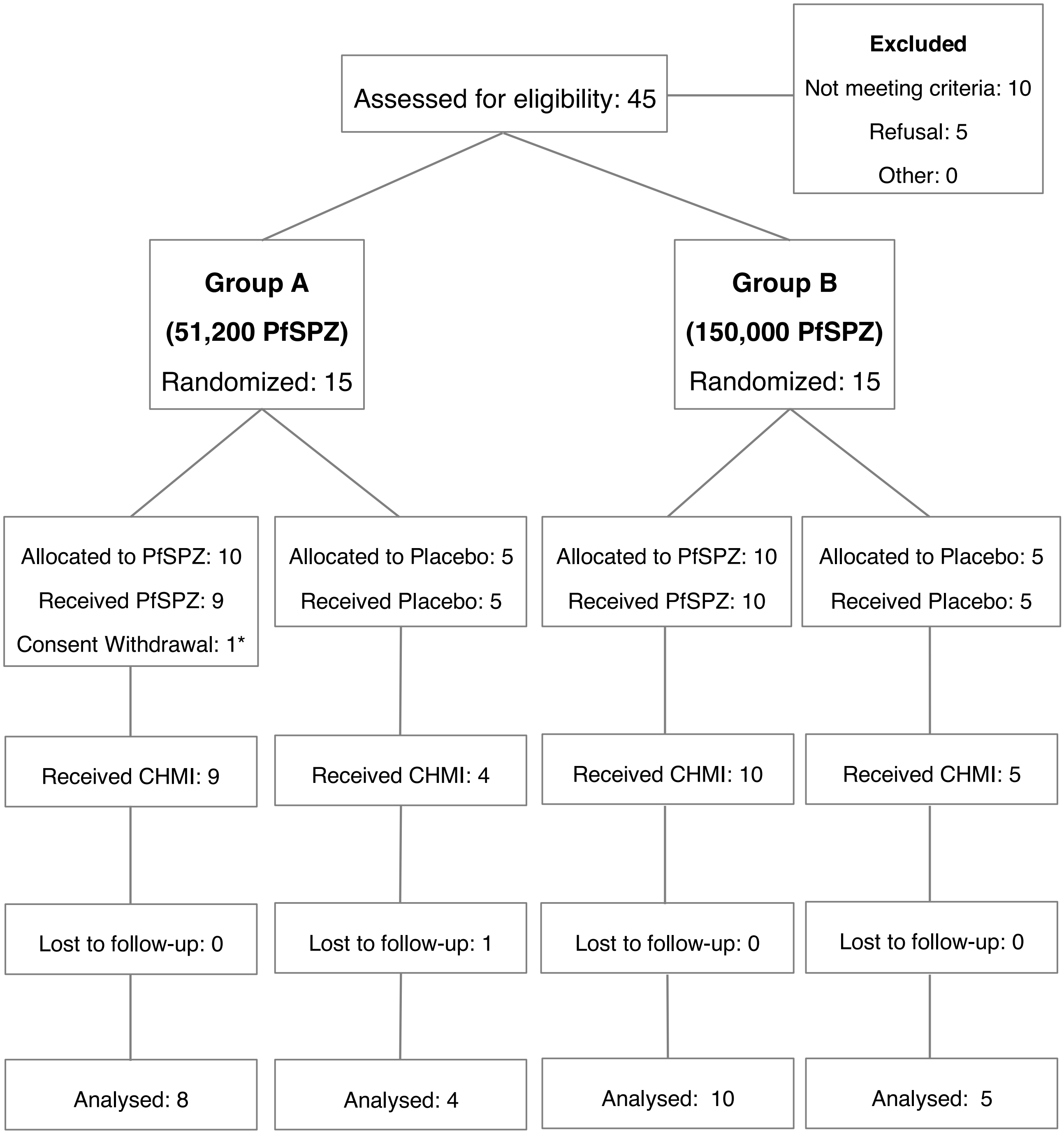

* Participant received only the first PfSPZ injection

Appendix Table S1: Baseline characteristics of study population.

	Vaccinees		Placebo
	$5.12x10^4$ PfSPZ	$1.5x10^5$ PfSPZ	
	n=10	n=10	n=10
**Sex**			
Female	7	6	5
Male	3	4	5
**Age (years)**			
Mean (± S.D.)	26.9 (± 6.8)	28.1 (± 2.2)	24.7 (± 4.6)
Median	24	28	24
Min, Max	19, 36	26, 32	19, 32
**BMI**			
under 18.5	1	1	1
18.5-24.9	7	5	6
25.0-29.9	2	4	3
30.0 or over	0	0	0
Mean (± SD)	22.4 (± 3.5)	22.2 (± 3.2)	24 (± 3.4)
Min, Max	18, 28	18, 27	18, 29

Appendix Table S2: Most frequent related adverse events.

	Placebo	Vaccinees	
		**$5.12x10^4$ PfSPZ**	**$1.5x10^5$ PfSPZ**
	n=10	n=10	n=10
**AEs related to immunization**	**33**	**19**	**13**
	Headache 21% (7)<br>• grade 1 (6)<br>• grade 2 (1)<br>Dizziness 12% (4)<br>• grade 1 (4)<br>Fatigue 5% (3)<br>• grade 1(3)<br>Tachycardia 5% (3)<br>• grade 1 (3)<br>Miscellaneous (48%)	Hypertension 31% (6)<br>• grade 1 (3)<br>• grade 2 (2)<br>• grade 3 (1)<br>Dizziness 21% (4)<br>• grade 1 (4)<br>Pruritus at injection site 10.5% (2)<br>• grade 1 (2)<br>Headache 10.5% (2)<br>• grade 1 (1)<br>• grade 2(1)<br>Diarrhoea 10.5% (2)<br>• grade 1 (2)<br>Miscellaneous (16%)	Fatigue 23% (3)<br>• grade 1 (2)<br>• grade 2 (1)<br>Neutropenia 30% (4)<br>• grade 1 (3)<br>• grade 2 (1)<br>Miscellaneous (46%)

**AEs related to CHMI**	**75**	**44**	**37**
	Lymphopenia 13% (10)<br>• grade 1 (5)<br>• grade 2 (4)<br>• grade 3 (1)<br>Fever/Feverish 9% (7)<br>• grade 1 (2)<br>• grade 2 (4)<br>• grade 3 (1)<br>Headache 12% (9)<br>• grade 1 (6)<br>• grade 2 (3)<br>Fatigue 9% (7)<br>• grade 1 (5)<br>• grade 2 (2)<br>Nausea 5% (4)<br>• grade 1 (4)<br>Sweating 5% (4)<br>• grade 1 (3)<br>• grade 2 (1)<br>Miscellaneous (54.6%)	Headache 16% (7)<br>• grade 1 (5)<br>• grade 2 (2)<br>Lymphopenia 9% (4)<br>• grade 1 (2)<br>• grade 2 (2)<br>Fatigue 9% (4)<br>• grade 1 (3)<br>• grade 2 (1)<br>Diarrhoea 7% (3)<br>• grade 1 (3)<br>Miscellaneous (59%)	Headache 16% (6)<br>• grade 1 (4)<br>• grade 2 (2)<br>Neutropenia 16% (6)<br>• grade 1 (4)<br>• grade 2 (2)<br>Fatigue 16% (6)<br>• grade 1 (5)<br>• grade 2 (1)<br>Lymphopenia 13% (5)<br>• grade 1 (3)<br>• grade 2 (2)<br>Miscellaneous (38%)

Appendix Table S3: Differentially recognized antigens, expression profiles and inclusion into subunit vaccine strategies

				mRNA expression [1-6]			Protein expression [4-8]			
Protein	Gene ID	Description	Surface	SPZ	LS	BS	SPZ	LS	BS	Subunit vaccine
Liver stage antigen 1 (LSA1)	PF3D7_1036400	liver stage antigen	-	-	-	-	-	+ [9]	-	yes [10]
Liver specific protein 2 (LISP2)	PF3D7_0405300	exported to hepatocyte	-	-	+	-	-	+	-	-
Merozoite surface protein 5 (MSP5)	PF3D7_0206900	duplicated antigen	yes	+	-	+	+	-	+	yes [11]
Circumsporozoite protein (CSP)	PF3D7_0304600	sporozoite surface, yes dominant antigen	+	-	-		+	-	-	yes [12]
Repetitive interspersed families of polypeptides (Rifin; RIF)	PF3D7_1040800	variant antigen	?	+	-	-	+	-	-	-
Erythrocyte binding antigen 181 (EBA181)	PF3D7_0102500	parasite invasion	yes	+	+	+	+	+	+	-
Parasitophorous vacuole membrane protein S16 (Pfs16)	PF3D7_0406200	sexual stage/sporozoite-specific antigen	yes	+	-	+	+	-	+	yes [13]
Dynactin subunit 2 (DCTN2/p50)	PF3D7_1346000	dynein anchor protein	-	+	-	-	+	-	-	-
Liver stage antigen 3 (LSA3)	PF3D7_0220000	ubiquitous antigen	-	+	+	+	+	+	+	yes [14]
Endoplasmin (GRP94/HSP90)	PF3D7_1222300	ER-resident chaperone	-	+	+	+	+	+	+	-
Reticulocyte binding protein 2 homologue b (Rh2b)	PF3D7_1335300	merozoite invasion yes	-	+	+	+	-	+	+	yes [15]
hypothetical protein	PF3D7_0706100	-	?	+	-	+	+	-	-	-

Blue and red denote antigens enriched in PfSPZ-CVac (CQ) and PfSPZ-CVac (AP) immune sera, respectively.

SPZ, salivary gland sporozoites; LS, liver stages; BS, asexual blood stages.

References:

1. Kappe SHI, Gardner MJ, Brown SM, *et al.* (2001) Exploring the transcriptome of the malaria sporozoite stage. ***Proc Natl Acad Sci USA*** **98**: 9895-9900.

2. Otto TD, Wilinski D, Assefa S, *et al.* (2010) New insights into the blood-stage transcriptome of *Plasmodium falciparum* using RNA-Seq. ***Mol Microbiol*** **76**: 12-24.

3. Otto TD, Böhme U, Jackson AP, *et al.* (2014) A comprehensive evaluation of rodent malaria parasite genomes and gene expression. ***BMC Biol*** **12**: 86.

4. Le Roch KG, Johnson JR, Florens L *et al.* (2004) Global analysis of transcript and protein levels across the *Plasmodium falciparum* life cycle. ***Genome Res*** **14**: 2308-2318.

5. Hall N, Karras M, Jaine RD, et al. (2005). A comprehensive survey of the *Plasmodium* life cycle by genomic, transcriptomic, and proteomic analyses. ***Science*** **307**: 82-86.

6. Tarun AS, Peng X, Dumpit RF, *et al.* (2008) A combined transcriptome and proteome survey of malaria parasite liver stages. ***Proc Natl Acad Sci USA*** **105**: 305-310.

7. Florens L, Washburn MP, Raine JD, *et al.* (2002) A proteomic view of the *Plasmodium falciparum* life cycle. ***Nature*** **419**: 520-526.

8. Lindner SE, Swearingen KE, Harupa A, et al. (2013) Total and putative surface proteomics of malaria parasite salivary gland sporozoites. ***Mol Cell Proteomics*** **12**: 1127-1143.

9. Guerin-Marchand C, Druilhe P, Galey B, *et al.* (1987) A liver-stage specific antigen of *Plasmodium falciparum* characterized by gene cloning. ***Nature*** **329**: 164-167.

10. Longley RJ, Salman AM, Cottingham MG, *et al.* (2015) Comparative assessment of vaccine vectors encoding ten malaria antigens identifies two protective liver-stage candidates. ***Sci Rep*** **5**: 11820.

11. Kedzierski L, Black CG, Coppel RL (2000) Immunization with recombinant *Plasmodium yoelii* merozoite surface protein 4/5 protects mice against lethal challenge. ***Infect Immun*** **68**: 6034-6037.

12. RTS,S clinical trials partnership (2015) Efficacy and safety of RTS,S/AS01 malaria vaccine with or without a booster dose in infants and children in Africa: final results of a phase 3, individually randomised, controlled trial. ***Lancet*** **386**: 31-45.

13. Moelans IID, Cohen J, Marchand M, *et al.* (1995) Induction of *Plasmodium falciparum* sporozoite-neutralzing antibodies upon vaccination with recombinant Pfs16 vaccinia virus and/or recombinant Pfs16 protein produced in yeast. ***Mol Biochem Parasitol*** **72**: 179-192.

14. Daubersies P, Thomas AW, Millet P, *et al.* (2000) Protection against *Plasmodium falciparum* malaria in chimpanzees by immunization with the conserved preerythrocytic liver-stage antigen 3. ***Nat Med*** **6**: 1258-1263.

15. Triglia T, Chen L, Lopaticki S, et al. (2011) *Plasmodium falciparum* merozoite invasion is inhibited by antibodies that target PfRh2a and b binding domains. ***PLoS Path*** **7**: e1002075.

Appendix Table S4: Numbers

Figure 1A

Images of liver stages in cultured hepatoma cells

group	untreated
n	2

one experiment, technical replicates, 2 wells per group

Figure 1B

% blood infection-negative mice

group	control
n	5

Figure 1C

relative levels *Pb*18s rRNA

group	atovaquone
n	5

one set of experiments

Figure 1D

relative level *Pb*18s rRNA

group	atovaquone-proguanil
n	6

one set of experiments

Figure 2A

% blood infection-negative mice

group	control
n	14

Figure 2B

relative level *Pb*18s rRNA

group	control
n	6

one set of experiments

Figure 2C

% IFNγ^{+} CD11a^{hi} CD8^{+} T cells

group	control
n	6

biological replicates; one set of experiments;
this experiment also generated data for EV4 A-E

Figure 2D

Reciprocal antibody titers

group	control
n	5

biological replicates; one set of experiments

Figure EV1A

Images of liver stages in cultured hepatoma cells

group	WT-irradiated
n	3

one set of experiments, technical replicates (3 wells per group)

Figure EV1B

Liver stage volume in % of untreated

group	untreated
n	41

one set of experiments, same as in 1A, technical replicates (2 wells per group)

Figure EV1C

Parasite numbers

group	untreated
n	2

one set of experiments, same as in 1A, technical replicates (2 wells per group)

Figure EV2A

% blood infection-negative mice

group	atovaquone-proguanil *ex vivo*
n	5

biological replicates; one set of experiments

Figure EV2B

% blood infection-negative mice

group	atovaquone-proguanil treated
n	5

biological replicates; one set of experiments

Figure EV2C

Parasitemia

group	atovaquone-proguanil treated
n	5

biological replicates; one set of experiments

Figure EV4A

No. of $CD8^+$ $CD62L^-$ T cells

group	control
n	6 (18)

one set of experiment, same as EV4 B-E and Fig 2C; biological, (technical) replicates

Figure EV4B

TRAP No. of IFNy in $CD8^+CD11a^{hi}$ cells

group	control
n	6

biological replicates; one set of experiments

Figure EV4C

S20 No. of IFNy in $CD8^+CD11a^{hi}$ cells

group	control
n	6

biological replicates; one set of experiments

Figure EV4D

S20 % of IFNy in $CD8^+CD11a^{hi}$ cells

group	control
n	6

biological replicates; one set of experiments

Figure EV 4 E

no peptide control % of IFNy in $CD8^+CD11a^{hi}$cells

group	control
n	6

biological replicates; one set of experiments

Appendix Table S5: Exact levels of all *p* values

Figure	Groups		Significant?	P-value		Test
Figure 1B						
	Kaplan Meier estimator	control *vs.* atovaquone	Yes	****	<0,0001	Log rank (Mantel-Cox) test
	Kaplan Meier estimator	control *vs.* atovaquone-proguanil	Yes	****	<0,0001	Log rank (Mantel-Cox) test
Figure 1C						
	Relative levels of *Pb*18s rRNA	control *vs.* atovaquone	Yes	**	0,0079	Mann-Whitney U test
Figure 1D						
	Relative levels of *Pb*18s rRNA	control *vs.* atovaquone-proguanil	Yes	**	0,0022	Mann-Whitney U test
Figure 2A						
	Kaplan Meyer estimator	control *vs.* atovaquone 3x	Yes	****	<0,0001	Log rank (Mantel-Cox) test
		control *vs.* atovaquone 2x	Yes	****	<0,0001	Log rank (Mantel-Cox) test
		control *vs.* atovaquone-proguanil	Yes	****	<0,0001	Log rank (Mantel-Cox) test
Figure 2B						
	Relative levels of *Pb*18s rRNA	control *vs.* atovaquone-proguanil	Yes	**	0,0043	Mann-Whitney U test
	Relative levels of *Pb*18s rRNA	control *vs.* atovaquone 3x	Yes	**	0,0022	Mann-Whitney U test
	Relative levels of *Pb*18s rRNA	control *vs.* atovaquone 2x	Yes	*	0,0159	Mann-Whitney U test
Figure 2C						
	% $IFN\gamma^{+}$ $CD11a^{hi}$ $CD8^{+}$ T-cells	control *vs.* atovaquone 3x	Yes	**	0,0022	Mann-Whitney U test
		control *vs.* atovaquone-proguanil 2x	Yes	**	0,0043	Mann-Whitney U test
Figure 2D						
	Reciprocal antibody titers	control *vs.* atovaquone 3x	Yes	**	0,0043	Mann-Whitney U test
		control *vs.* atovaquone-proguanil 2x	Yes	**	0,0079	Mann-Whitney U test
Figure EV1B						
	Liver stage volume in % of untreated	control *vs.* atovaquone	Yes	****	<0,0001	Mann-Whitney U test
		control *vs.* atovaquone-proguanil	Yes	****	<0,0001	Mann-Whitney U test

Figure EV1C						
	Parasite numbers	control *vs.* atovaquone	No	n.s.	0,6667	Mann-Whitney U test
		control *vs.* atovaquone-proguanil	No	n.s.	0,3333	Mann-Whitney U test
Figure EV2A						
	Kaplan Meyer estimator					
		control *vs.* atovaquone-*proguanil ex vivo*	No	n.s.	0.1967	Log rank (Mantel-Cox) test
Figure EV2B						
	Kaplan Meyer estimator					
		control vs. atovaquone-proguanil treated	No	n.s.	0.9999	Log rank (Mantel-Cox) test
Figure EV4A						
	No. of $CD8^+$ $CD62L^-$ T cells	control *vs.* atovaquone 3x	Yes	****	<0,0001	Mann-Whitney U test
		control *vs.* atovaquone-proguanil 2x	Yes	****	<0,0001	Mann-Whitney U test
Figure EV4B						
	TRAP No. of IFNy in $CD8^+CD11a^{hi}$ cells	control *vs.* atovaquone 3x	Yes	**	0,0022	Mann-Whitney U test
		control *vs.* atovaquone-proguanil 2x	Yes	**	0,0043	Mann-Whitney U test
Figure EV4C						
	S20 No. of IFNy in $CD8^+CD11a^{hi}$ cells	control *vs.* atovaquone 3x	Yes	**	0,0043	Mann-Whitney U test
		control *vs.* atovaquone-proguanil 2x	Yes	**	0,0043	Mann-Whitney U test
Figure EV4D						
	S 20 % of IFNy in $CD8^+CD11a^{hi}$ cells	control *vs.* atovaquone 3x	Yes	**	0,0022	Mann-Whitney U test
		control *vs.* atovaquone-proguanil 2x	Yes	**	0,0043	Mann-Whitney U test
Figure EV4E						
	No peptide control % of IFNy in $CD8^+CD11a^{hi}$ cells	control *vs.* atovaquone 3x	No	n.s.	0,6991	Mann-Whitney U test
		control *vs.* atovaquone-proguanil 2x	No	n.s.	0,5346	Mann-Whitney U test
