# Supplementary figures and images for: Complete attenuation of Plasmodium falciparum sporozoites by atovaquone–proguanil
Source: EMBO Mol Med. 2025 Sep 29;17(11):2875–900. doi: 10.1038/s44321-025-00301-8 (PMC12602697; doi:10.1038/s44321-025-00301-8)

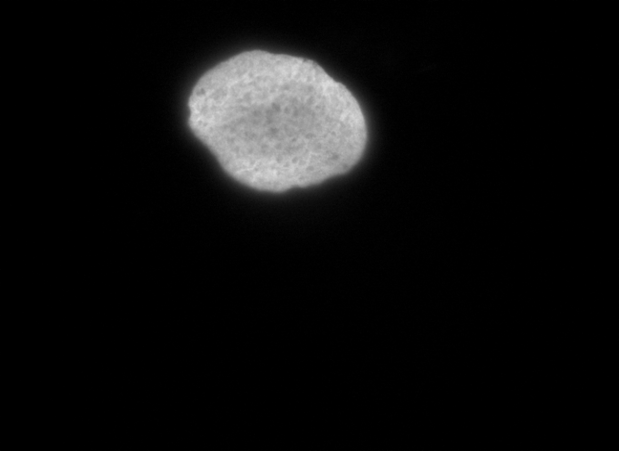

Supplement: Supplementary file 3 — Source data Fig. 1 [file 44321_2025_301_MOESM3_ESM.zip › Figure 1/1A/Figure 1 A WT anti HSP70 antibody.png]

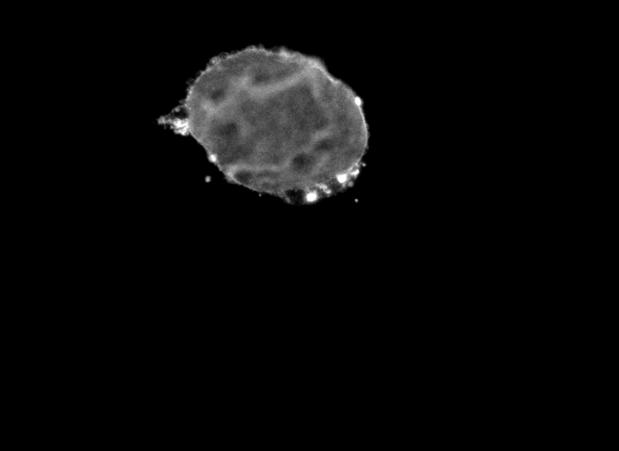

Supplement: Supplementary file 3 — Source data Fig. 1 [file 44321_2025_301_MOESM3_ESM.zip › Figure 1/1A/Figure 1 A WT anti UIS4 antibody.png]

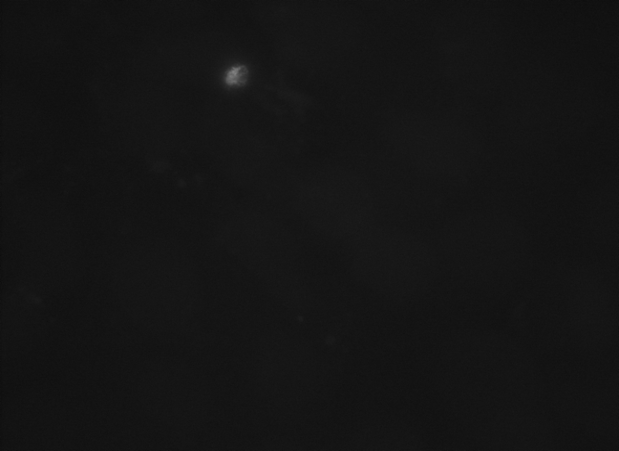

Supplement: Supplementary file 3 — Source data Fig. 1 [file 44321_2025_301_MOESM3_ESM.zip › Figure 1/1A/Figure 1 A Atovaquone anti HSP70 antibody.png]

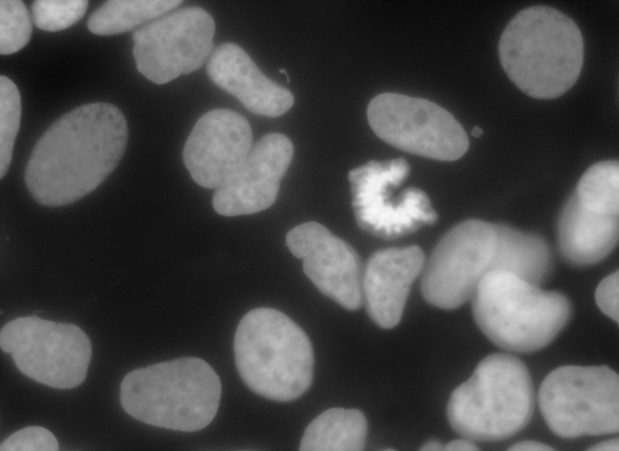

Supplement: Supplementary file 3 — Source data Fig. 1 [file 44321_2025_301_MOESM3_ESM.zip › Figure 1/1A/Figure 1 A Atovaquone-Proguanil nuclear stain.png]

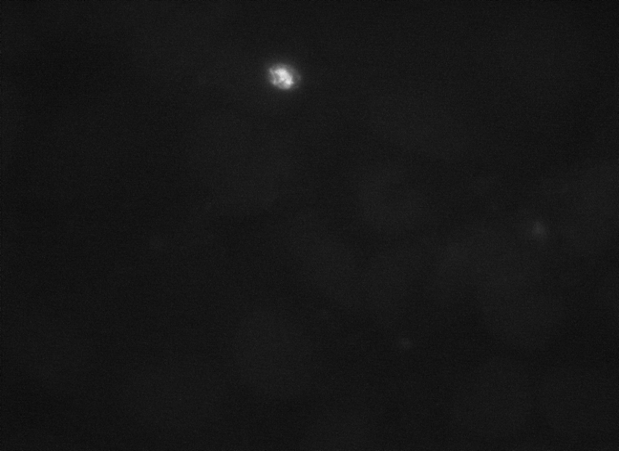

Supplement: Supplementary file 3 — Source data Fig. 1 [file 44321_2025_301_MOESM3_ESM.zip › Figure 1/1A/Figure 1 A Atovaquone-Proguanil anti HSP70 antibody.png]

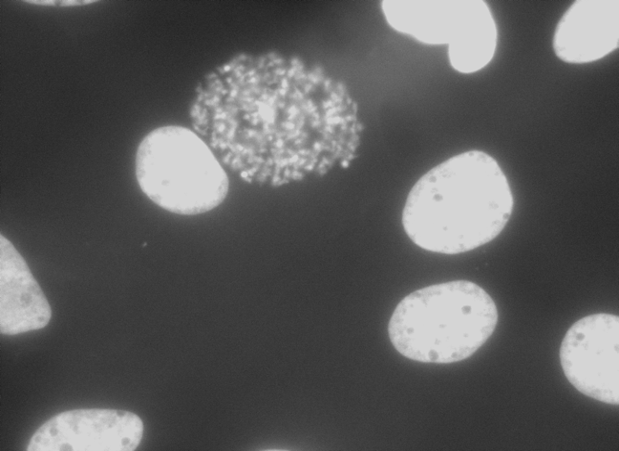

Supplement: Supplementary file 3 — Source data Fig. 1 [file 44321_2025_301_MOESM3_ESM.zip › Figure 1/1A/Figure 1 A WT nuclear stain.png]

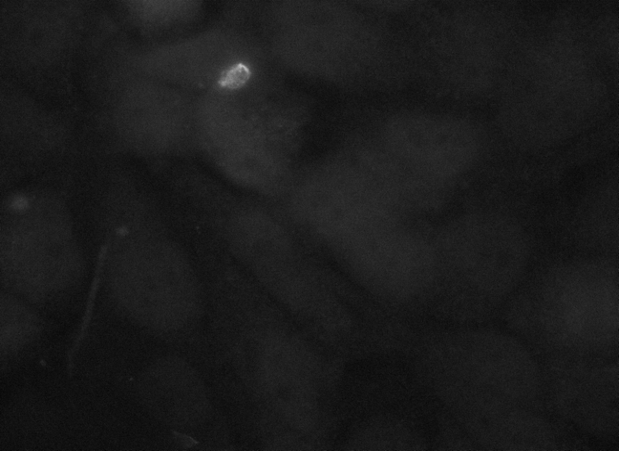

Supplement: Supplementary file 3 — Source data Fig. 1 [file 44321_2025_301_MOESM3_ESM.zip › Figure 1/1A/Figure 1 A Atovaquone anti UIS4 antibody.png]

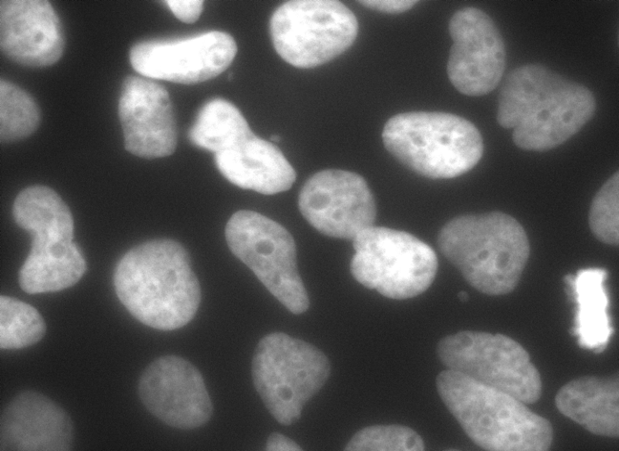

Supplement: Supplementary file 3 — Source data Fig. 1 [file 44321_2025_301_MOESM3_ESM.zip › Figure 1/1A/Figure 1 A Atovaquone nuclear stain.png]

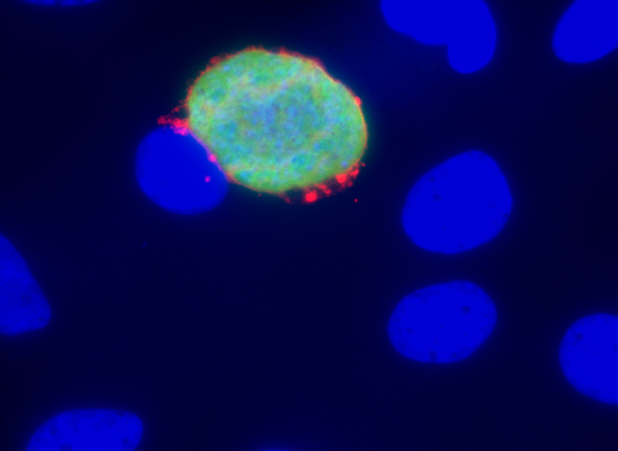

Supplement: Supplementary file 3 — Source data Fig. 1 [file 44321_2025_301_MOESM3_ESM.zip › Figure 1/1A/Figure 1 A WT merge.png]

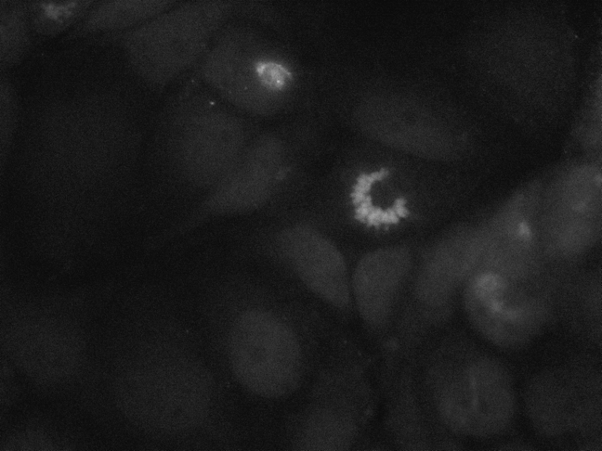

Supplement: Supplementary file 3 — Source data Fig. 1 [file 44321_2025_301_MOESM3_ESM.zip › Figure 1/1A/Figure 1 A Atovaquone-Proguanil anti UIS4 antibody.png]

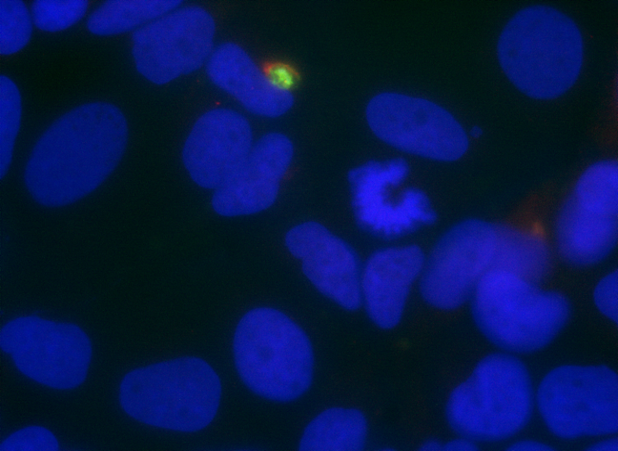

Supplement: Supplementary file 3 — Source data Fig. 1 [file 44321_2025_301_MOESM3_ESM.zip › Figure 1/1A/Figure 1 A Atovaquone-Proguanil merge.png]

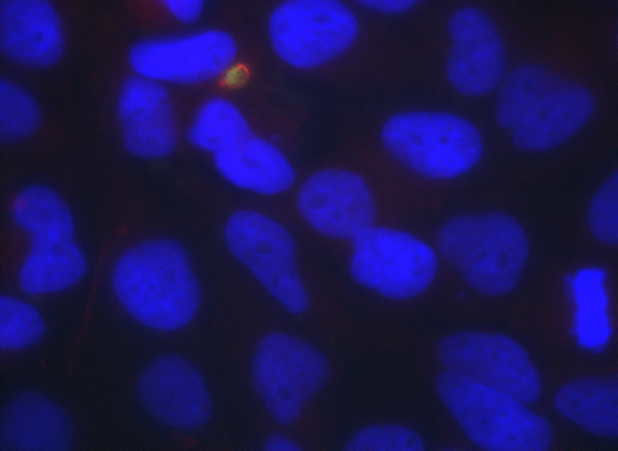

Supplement: Supplementary file 3 — Source data Fig. 1 [file 44321_2025_301_MOESM3_ESM.zip › Figure 1/1A/Figure 1 A Atovaquone merge.png]

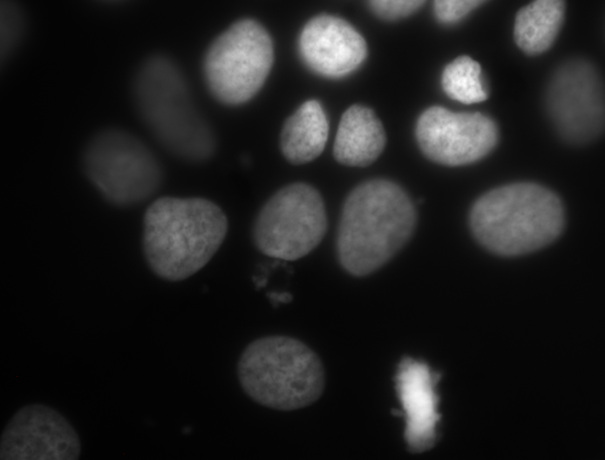

Supplement: Supplementary file 8 — Figure EV1 Source Data [file 44321_2025_301_MOESM8_ESM.zip › Figure EV1/EV1A/WT irradiated 116h nuclear stain.png]

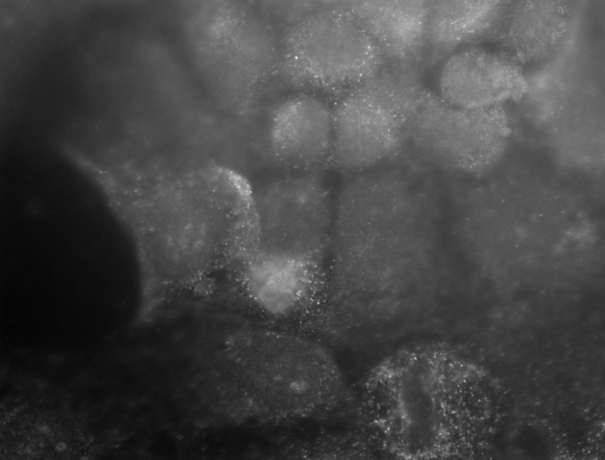

Supplement: Supplementary file 8 — Figure EV1 Source Data [file 44321_2025_301_MOESM8_ESM.zip › Figure EV1/EV1A/WT irradiated 116h anti UIS4.png]

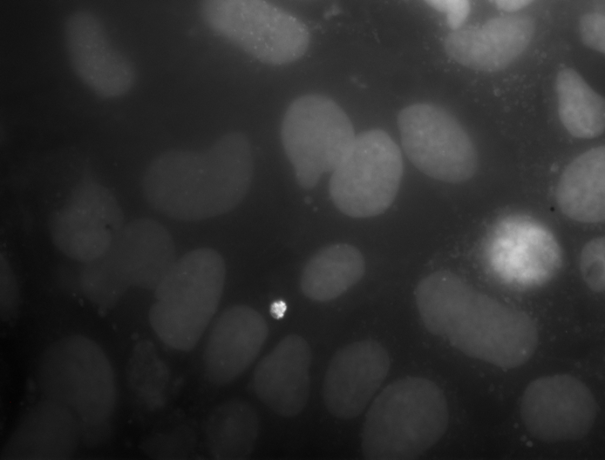

Supplement: Supplementary file 8 — Figure EV1 Source Data [file 44321_2025_301_MOESM8_ESM.zip › Figure EV1/EV1A/WT irradiated 96h anti HSP70.png]

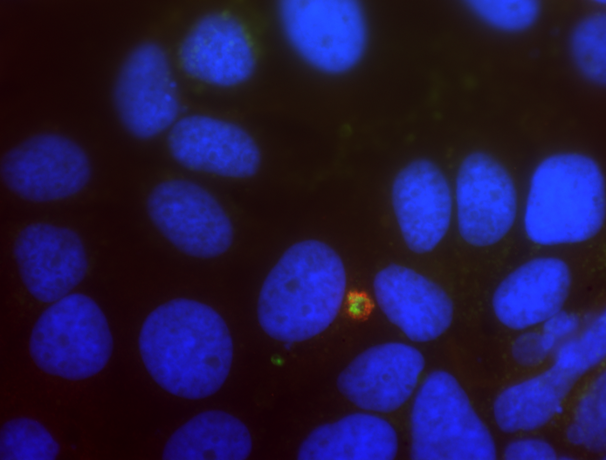

Supplement: Supplementary file 8 — Figure EV1 Source Data [file 44321_2025_301_MOESM8_ESM.zip › Figure EV1/EV1A/WT irradiated 48h merge.png]

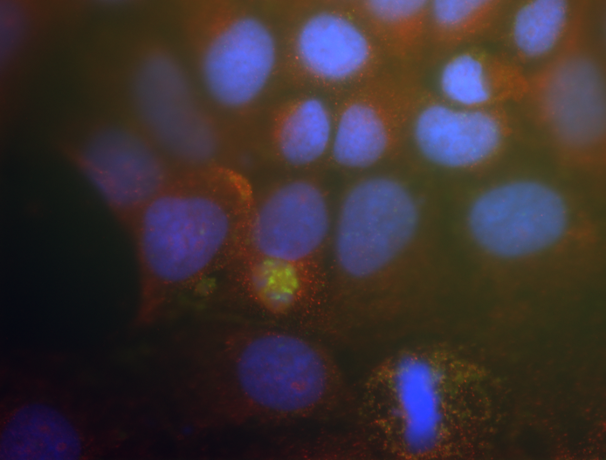

Supplement: Supplementary file 8 — Figure EV1 Source Data [file 44321_2025_301_MOESM8_ESM.zip › Figure EV1/EV1A/WT irradiated 116h merge.png]

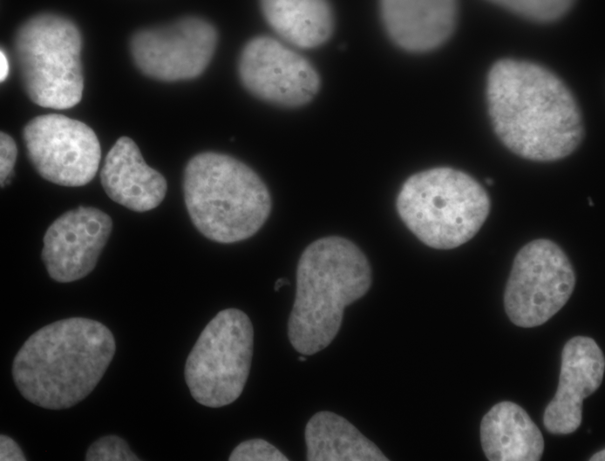

Supplement: Supplementary file 8 — Figure EV1 Source Data [file 44321_2025_301_MOESM8_ESM.zip › Figure EV1/EV1A/Atovaquone-Proguanil 48h nuclear stain.png]

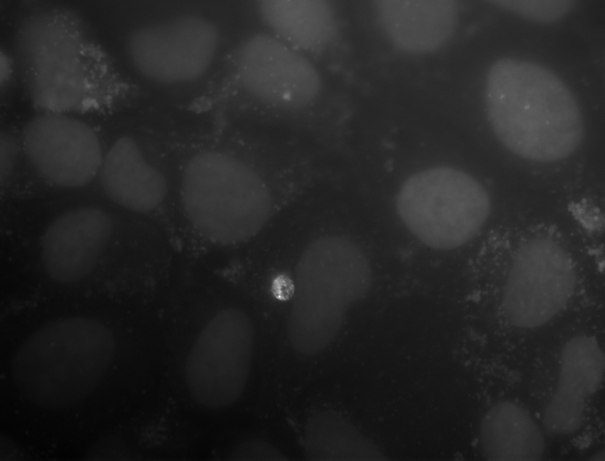

Supplement: Supplementary file 8 — Figure EV1 Source Data [file 44321_2025_301_MOESM8_ESM.zip › Figure EV1/EV1A/Atovaquone-Proguanil 48h anti HSP70.png]

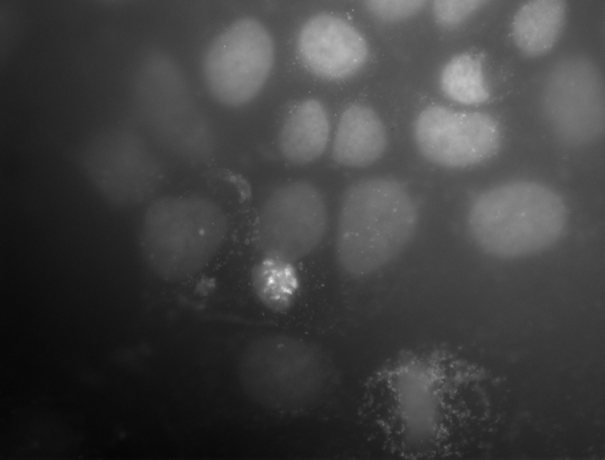

Supplement: Supplementary file 8 — Figure EV1 Source Data [file 44321_2025_301_MOESM8_ESM.zip › Figure EV1/EV1A/WT irradiated 116h anti HSP70.png]

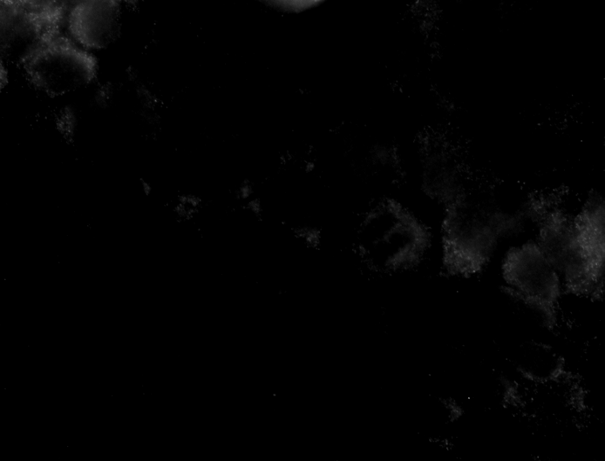

Supplement: Supplementary file 8 — Figure EV1 Source Data [file 44321_2025_301_MOESM8_ESM.zip › Figure EV1/EV1A/Atovaquone-Proguanil 96h anti UIS4.png]

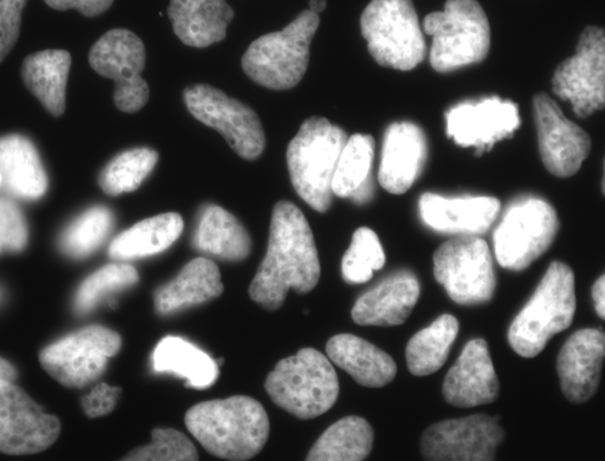

Supplement: Supplementary file 8 — Figure EV1 Source Data [file 44321_2025_301_MOESM8_ESM.zip › Figure EV1/EV1A/Atovaquone-Proguanil 116h nuclear stain.png]

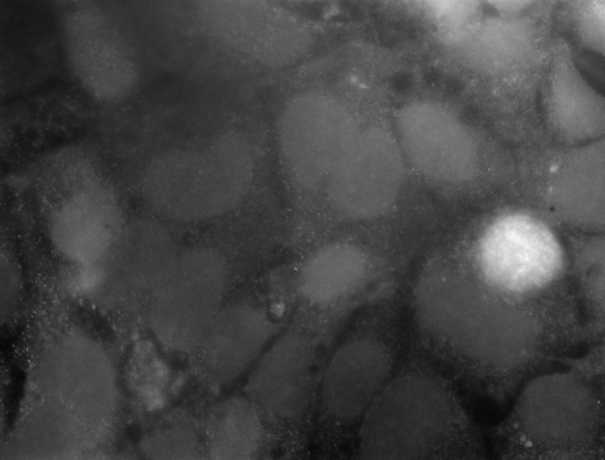

Supplement: Supplementary file 8 — Figure EV1 Source Data [file 44321_2025_301_MOESM8_ESM.zip › Figure EV1/EV1A/WT irradiated 96h anti UIS4.png]

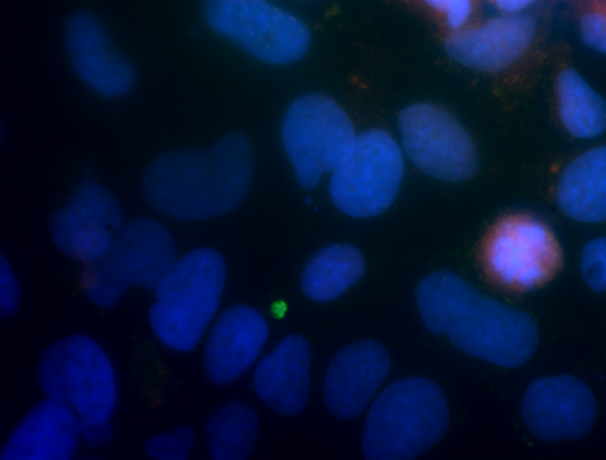

Supplement: Supplementary file 8 — Figure EV1 Source Data [file 44321_2025_301_MOESM8_ESM.zip › Figure EV1/EV1A/WT irradiated 96h merge.png]

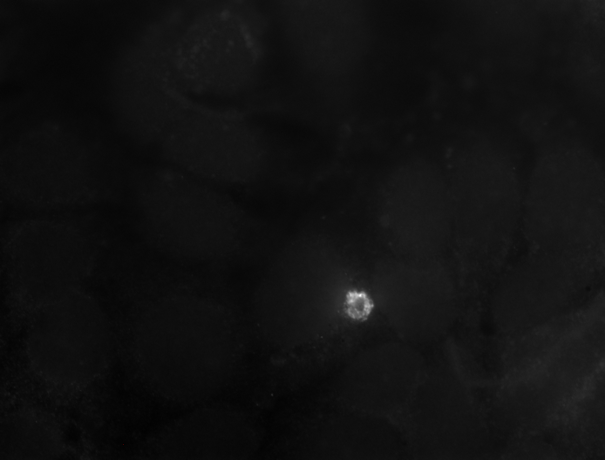

Supplement: Supplementary file 8 — Figure EV1 Source Data [file 44321_2025_301_MOESM8_ESM.zip › Figure EV1/EV1A/WT irradiated 48h anti UIS4.png]

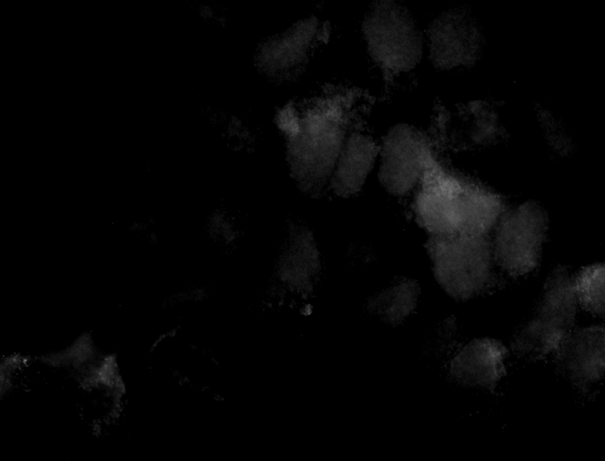

Supplement: Supplementary file 8 — Figure EV1 Source Data [file 44321_2025_301_MOESM8_ESM.zip › Figure EV1/EV1A/Atovaquone-Proguanil 116h anti UIS4.png]

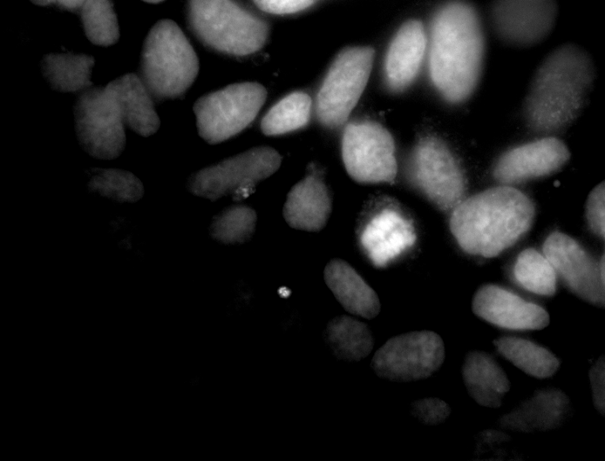

Supplement: Supplementary file 8 — Figure EV1 Source Data [file 44321_2025_301_MOESM8_ESM.zip › Figure EV1/EV1A/Atovaquone-Proguanil 96h anti HSP70.png]

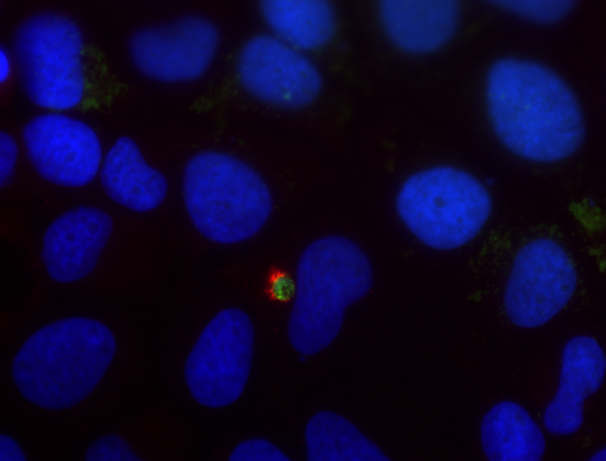

Supplement: Supplementary file 8 — Figure EV1 Source Data [file 44321_2025_301_MOESM8_ESM.zip › Figure EV1/EV1A/Atovaquone-Proguanil 48h merge.png]

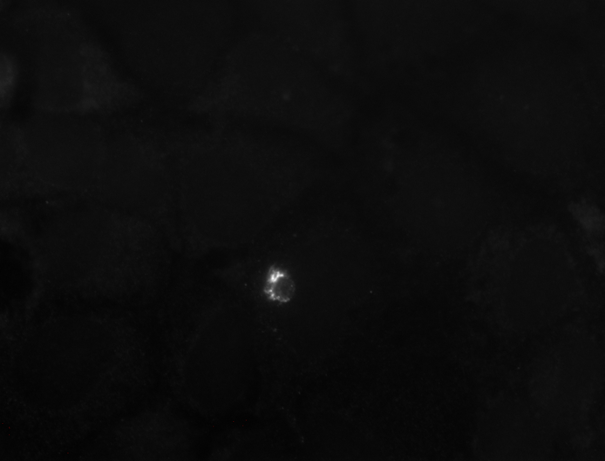

Supplement: Supplementary file 8 — Figure EV1 Source Data [file 44321_2025_301_MOESM8_ESM.zip › Figure EV1/EV1A/Atovaquone-Proguanil 48h anti UIS4.png]

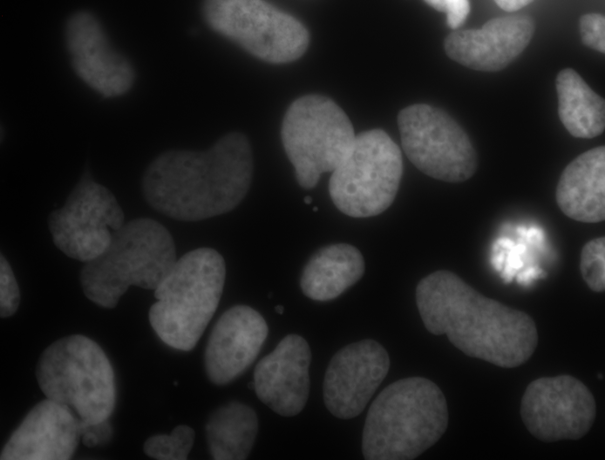

Supplement: Supplementary file 8 — Figure EV1 Source Data [file 44321_2025_301_MOESM8_ESM.zip › Figure EV1/EV1A/WT irradiated 96h nuclear stain.png]

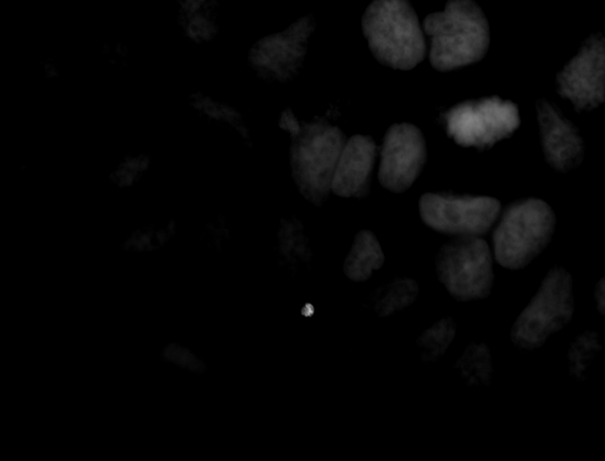

Supplement: Supplementary file 8 — Figure EV1 Source Data [file 44321_2025_301_MOESM8_ESM.zip › Figure EV1/EV1A/Atovaquone-Proguanil 116h anti HSP70.png]

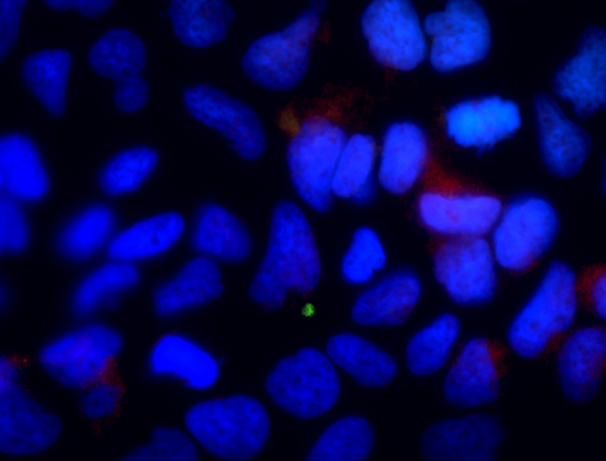

Supplement: Supplementary file 8 — Figure EV1 Source Data [file 44321_2025_301_MOESM8_ESM.zip › Figure EV1/EV1A/Atovaquone-Proguanil 116h merge.png]

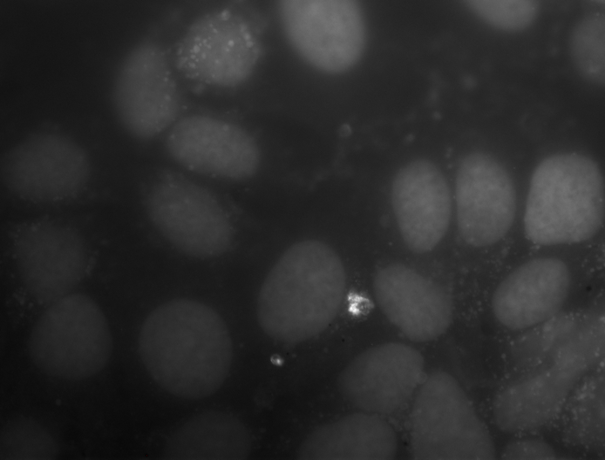

Supplement: Supplementary file 8 — Figure EV1 Source Data [file 44321_2025_301_MOESM8_ESM.zip › Figure EV1/EV1A/WT irradiated 48h anti HSP70.png]

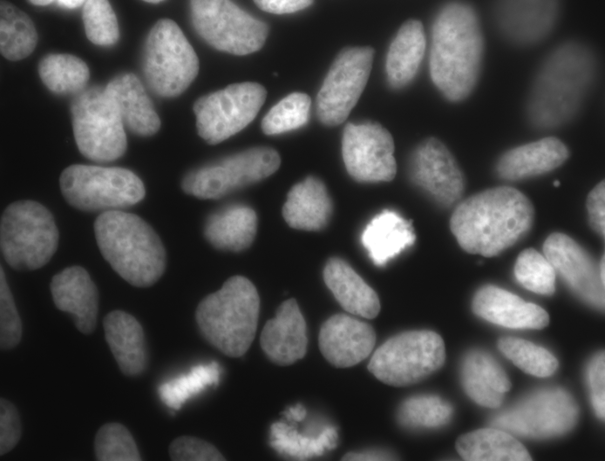

Supplement: Supplementary file 8 — Figure EV1 Source Data [file 44321_2025_301_MOESM8_ESM.zip › Figure EV1/EV1A/Atovaquone-Proguanil 96h nuclear stain.png]

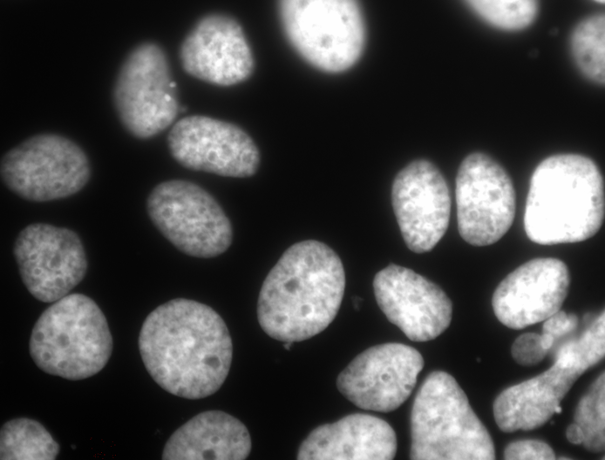

Supplement: Supplementary file 8 — Figure EV1 Source Data [file 44321_2025_301_MOESM8_ESM.zip › Figure EV1/EV1A/WT irradiated 48h nuclear stain.png]

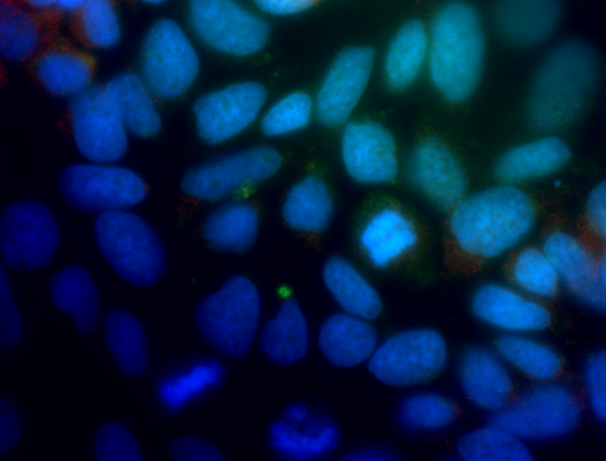

Supplement: Supplementary file 8 — Figure EV1 Source Data [file 44321_2025_301_MOESM8_ESM.zip › Figure EV1/EV1A/Atovaquone-Proguanil 96h merge.png]
