# Supplementary material for: Complete attenuation of Plasmodium falciparum sporozoites by atovaquone–proguanil
Source: EMBO Mol Med. 2025 Sep 29;17(11):2875–900. doi: 10.1038/s44321-025-00301-8 (PMC12602697; doi:10.1038/s44321-025-00301-8)
Supplement: Supplementary file 12 — Expanded View Figures [file 44321_2025_301_MOESM12_ESM.pdf]

Expanded View Figures

Figure EV1. *Plasmodium* liver stages treated with atovaquone–proguanil retain their invasive capacity, are fully arrested, and persist 5 days after infection in vitro.

(**A**) Composite fluorescence micrographs of mature *Plasmodium berghei* liver stages in cultured hepatoma cells. Shown are representative images of liver stages 48, 96, and 116 h after infection with sporozoites. During the first 3 h, cultures were exposed to atovaquone–proguanil. Irradiated, untreated sporozoites served as a control. Parasites were visualised by fluorescent staining of the cytoplasm (green; anti-*Pb*HSP70 antibody), the parasitophorous vacuole membrane (red; anti-*Pb*UIS4 anti-serum), and nuclei (blue; Hoechst 33342). Scale bars: 10 µm. (**B**) Quantification of liver stage volumes after prophylactic drug treatment. Parasite volume was quantified 48 h after infection and normalised to the average volume of untreated parasites. Shown are mean percentages (±S.D.). Atovaquone, ***$p < 0.0001$; atovaquone–proguanil, ***$p < 0.0001$; Mann–Whitney *U*-test. Colour code: white circles, untreated ($n = 41$); blue circles, atovaquone ($n = 44$); red circles, atovaquone–proguanil ($n = 41$). (**C**) Sporozoite invasion is unaffected by short-term drug treatment. Shown are mean numbers of liver stages (±S.D.) in cultured hepatoma cells 48 h after initial 3 h co-administration of live sporozoites and atovaquone or atovaquone–proguanil. Untreated sporozoites served as controls. ns, non-significant. Atovaquone, ns $p = 0.6667$; atovaquone–proguanil, ns $p = 0.3333$; Mann–Whitney *U*-test. Colour code: white bar, untreated ($n = 2$), blue bar, atovaquone ($n = 2$); red bar, atovaquone–proguanil ($n = 2$). All in vitro liver stage experiments were performed in two technical replicates. Data in (**A–C**) are from single biological experiments performed with two technical replicates. Number and nature of the replicates as well as exact *p* values are shown in Appendix Tables S4 and S5. Source data are available online for this figure.

▶

 © The Author(s)

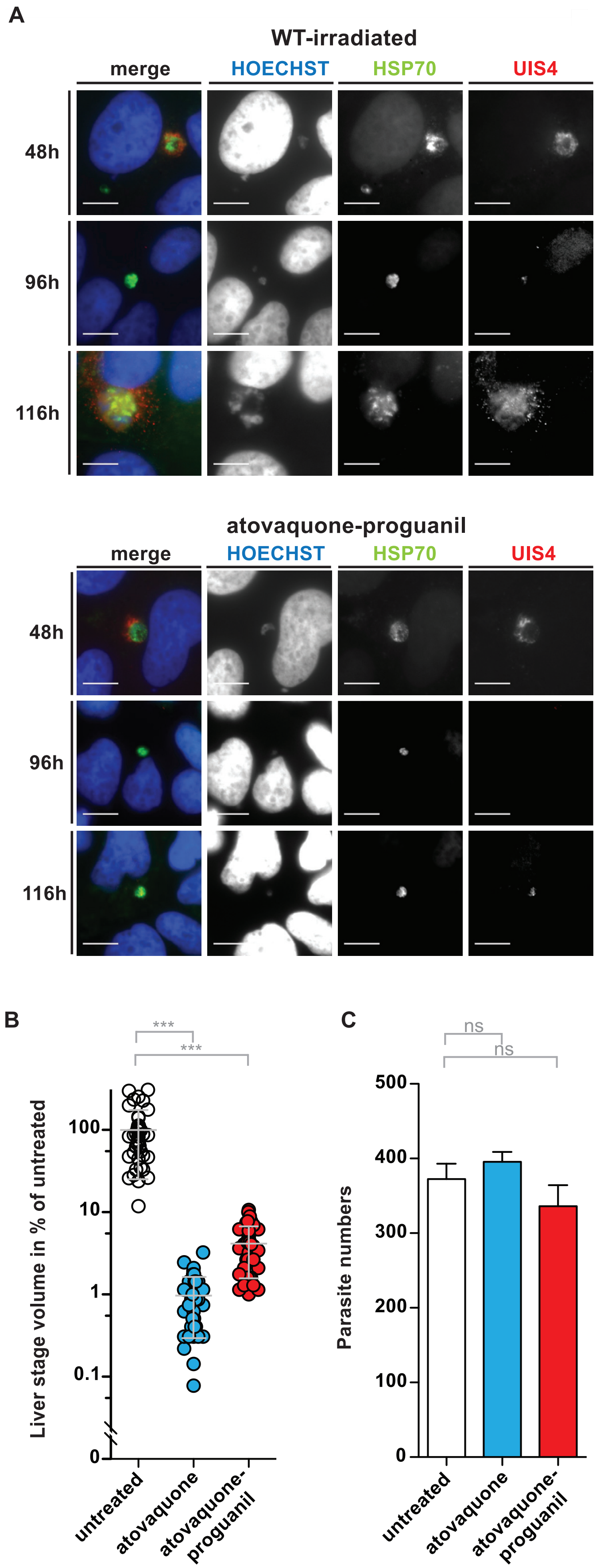
A
WT-irradiated
merge
HOECHST
HSP70
UIS4
48h
96h
116h
atovaquone-proguanil
merge
HOECHST
HSP70
UIS4
48h
96h
116h
B
***
***
Liver stage volume in % of untreated
100
10
1
0.1
0
untreated
atovaquone
atovaquone-proguanil
C
ns
ns
Parasite numbers
500
400
300
200
100
0
untreated
atovaquone
atovaquone-proguanil

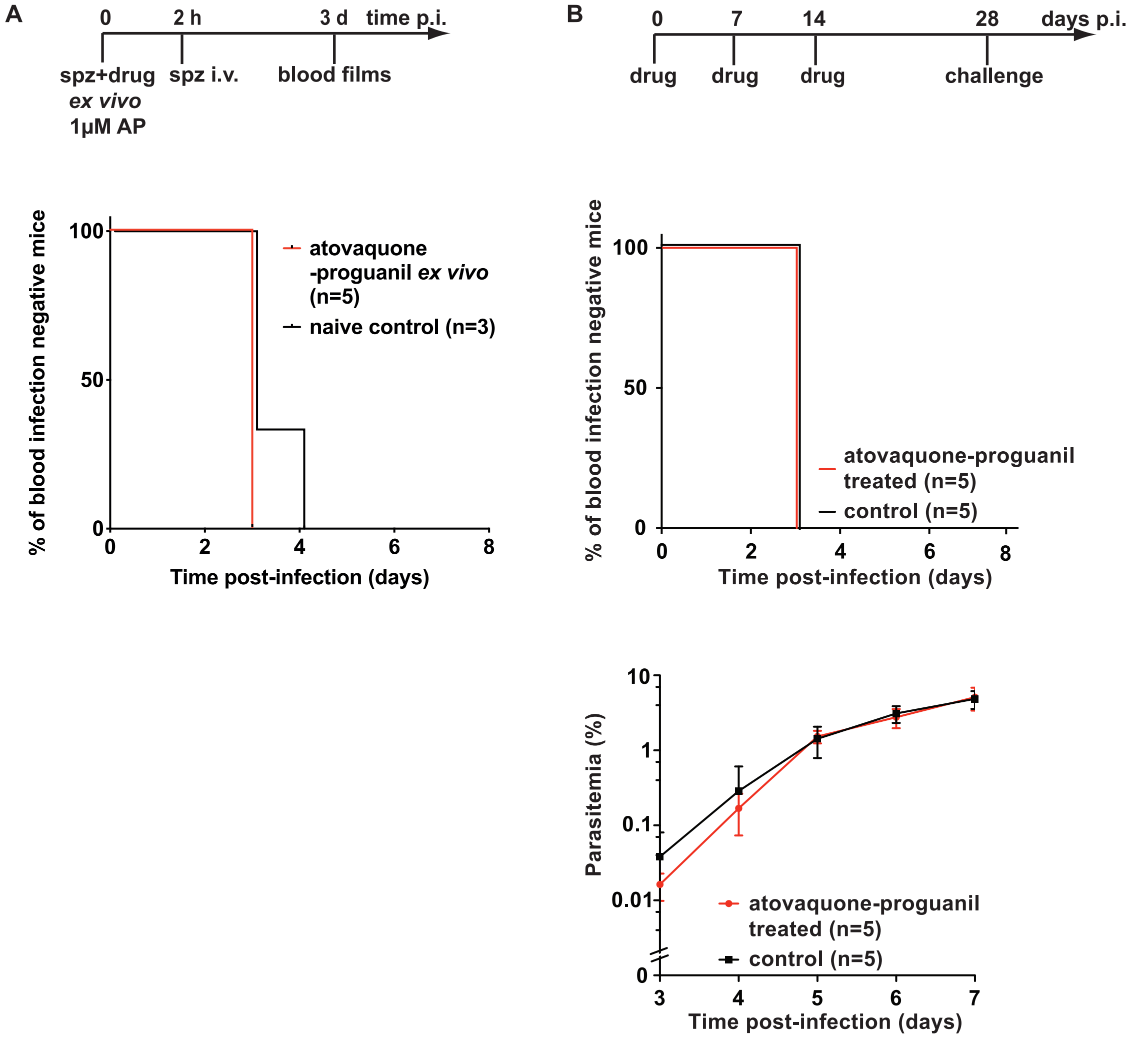

Figure EV2. Sporozoites exposed to AP retain infectivity and no interference from chemo-attenuation with atovaquone-proguanil with subsequent assessment of protection.

(**A**) Pre-exposure of Pb sporozoites to AP on ice for 2 h does not inhibit mouse infection. Shown are the study design (top) and Kaplan–Meier analysis of time to blood infection. ns, non-significant. Control vs. atovaquone ex vivo, ns $p = 0.1967$; log rank (Mantel–Cox) test. Colour code: black line, control ($n = 3$); red line, atovaquone–proguanil ($n = 5$). (**B**) Atovaquone–proguanil does not inhibit infections in naive mice when challenged 2 weeks after drug administration. C57BL/6 mice were treated three times at weekly intervals with 3/1.2 mg/kg atovaquone–proguanil (top). Kaplan–Meier analysis of time to blood infection upon challenge with 10^4 sporozoites (centre). ns, non-significant. Control vs. atovaquone-treated, ns $p > 0.99$; log rank (Mantel–Cox) test. Kinetics of blood stage infections after challenge (bottom). Parasitaemia was determined by daily microscopic examination of Giemsa-stained blood films. Shown are mean asexual blood stage parasite densities (±S.D.). Colour code: black line, control ($n = 5$); red line, atovaquone–proguanil ($n = 5$). Number and nature of the replicates as well as exact p values are shown in Appendix Tables S4 and S5. Source data are available online for this figure.

 © The Author(s)

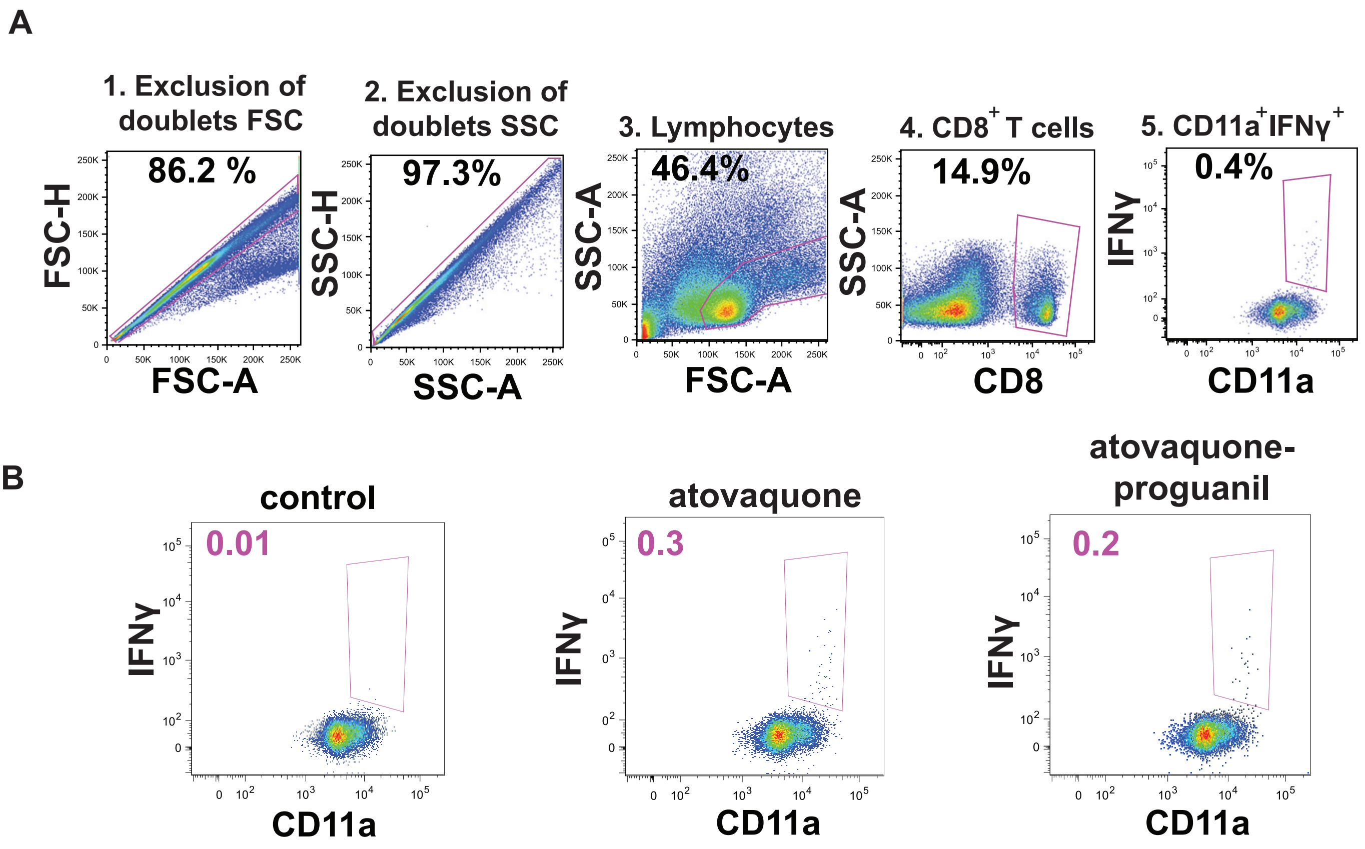

Figure EV3. Gating strategy for the identification of IFNγ-secreting activated CD8+ CD11a^hi T cells after re-stimulation with SSP2/TRAP$_{130\text{-}138}$ peptide.

(**A**) Shown are representative FACS plots illustrating the gating strategy for IFNγ secretion by CD8+ CD11a^hi T cells after re-stimulation. (**B**) Representative FACS plots of CD8+ CD11a^hi T cells positive for intracellular IFNγ expression after re-stimulation with the SSP2/TRAP$_{130\text{-}138}$ peptide. Numbers show the percentage of IFNγ produced by CD8+ CD11a^hi T cells.

© The Author(s)

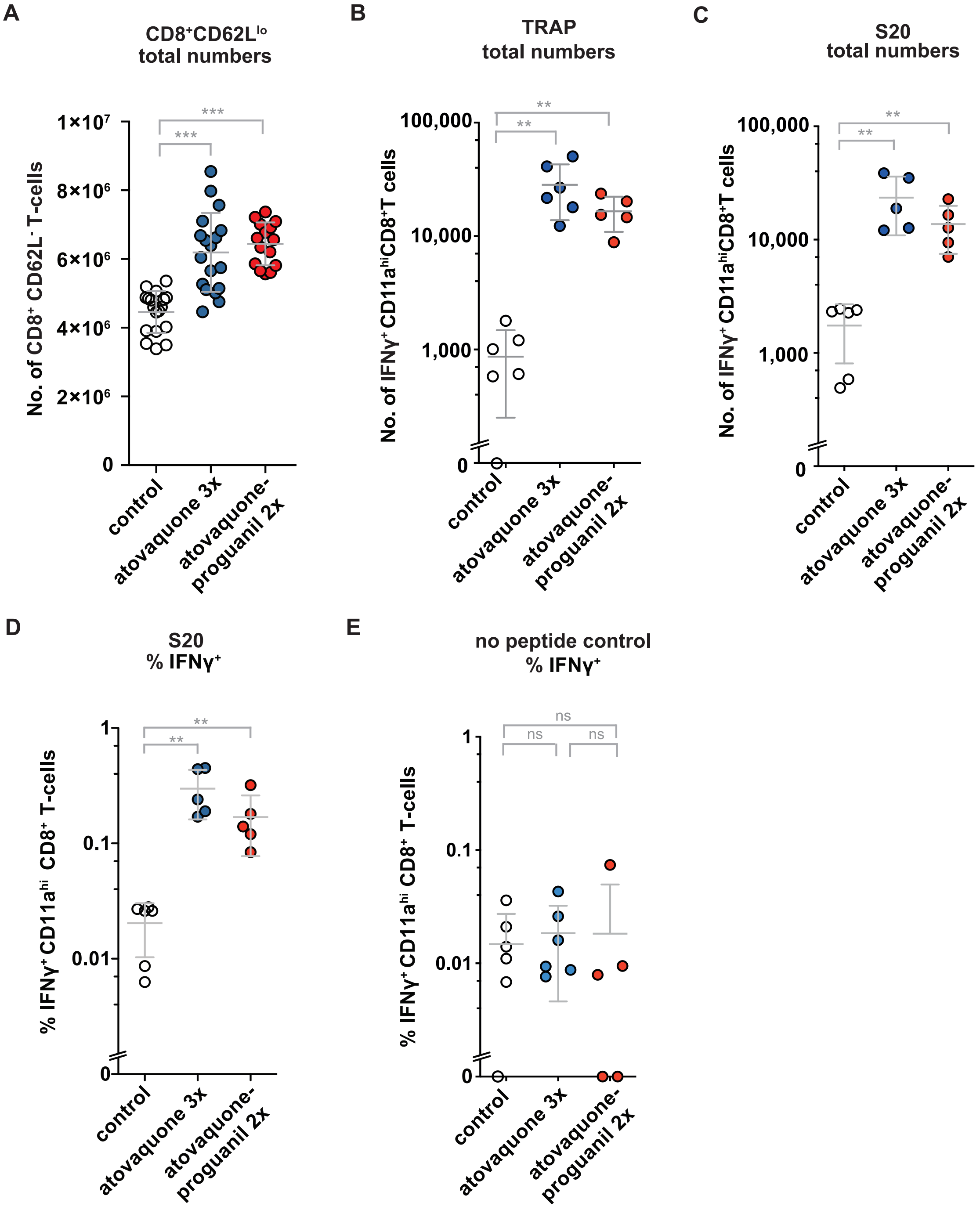
A
CD8+CD62Llo
total numbers
No. of CD8+ CD62L- T-cells
1×10^7
8×10^6
6×10^6
4×10^6
2×10^6
0
***
***
control
atovaquone 3x
atovaquone-proguanil 2x
B
TRAP
total numbers
No. of IFNγ+ CD11ahiCD8+T cells
100,000
10,000
1,000
0
**
**
control
atovaquone 3x
atovaquone-proguanil 2x
C
S20
total numbers
No. of IFNγ+CD11ahiCD8+T cells
100,000
10,000
1,000
0
**
**
control
atovaquone 3x
atovaquone-proguanil 2x
D
S20
% IFNγ+
% IFNγ+CD11ahi CD8+ T-cells
1
0.1
0.01
0
**
**
control
atovaquone 3x
atovaquone-proguanil 2x
E
no peptide control
% IFNγ+
% IFNγ+CD11ahi CD8+ T-cells
1
0.1
0.01
0
ns
ns
ns
control
atovaquone 3x
atovaquone-proguanil 2x

 © The Author(s)

Figure EV4. Effector memory T cells and proportion of SSP2/TRAP$_{130\text{-}138}$- and S20$_{318\text{-}326}$-experienced CD8$^+$ CD11a^{hi} T cells.

Mice were immunised by co-administration of sporozoites and a single dose of atovaquone (3 mg/kg i.p.) or atovaquone–proguanil (3/1.2 mg/kg i.p.). Mice were immunised three times (atovaquone co-administration) and twice (atovaquone–proguanil co-administration). Naive mice served as controls. Sporozoite challenge was done by i.v. injection of 10^4 sporozoites 3–4 weeks after the last immunisation. (**A**) Quantification of total CD8$^+$ CD62L^{lo} T cells from spleens of immunised or control mice. Shown are mean values (±S.D.). Control vs. atovaquone, ***$p < 0.0001$; control vs. atovaquone–proguanil, ***$p < 0.0001$; Mann–Whitney *U*-test. Colour code: white circles, control ($n = 6$); blue circles, atovaquone ($n = 6$); red circles, atovaquone–proguanil ($n = 5$). Cells were quantified in three technical replicates. (**B**) Quantification of numbers of SSP2/TRAP$_{130\text{-}138}$ peptide-specific IFNγ-secretion by CD8$^+$ CD11a^{hi} T cells from spleens of immunised or control mice. Shown are mean values (±S.D.). Control vs. atovaquone, **$p = 0.0022$; control vs. atovaquone–proguanil, **$p = 0.0043$; Mann–Whitney *U*-test. Colour code: white circles, control ($n = 6$); blue circles, atovaquone ($n = 6$); red circles, atovaquone–proguanil ($n = 5$). (**C**, **D**) Quantification of numbers (**C**) and percentage (**D**) of S20$_{318\text{-}326}$ peptide-specific IFNγ-secretion by CD8$^+$ CD11a^{hi} T cells from the spleens of immunised or control mice. Shown are mean values (±S.D.). (**C**) Control vs. atovaquone, **$p = 0.0043$; control vs. atovaquone–proguanil, **$p = 0.0043$. (**D**) Control vs. atovaquone, **$p = 0.0022$; control vs. atovaquone–proguanil, **$p = 0.0043$; Mann–Whitney *U*-test. Colour code: white circles, control ($n = 6$); blue circles, atovaquone ($n = 5$); red circles, atovaquone–proguanil ($n = 5$). (**E**) Quantification of the percentage of baseline IFNγ-secretion by CD8$^+$ CD11a^{hi} T cells without peptide stimulation. Shown are mean values (±S.D.). ns, non-significant. Control vs. atovaquone, ns $p = 0.6991$; control vs. atovaquone–proguanil, ns $p = 0.53$; Mann–Whitney *U*-test. Colour code: white circles, control ($n = 6$); blue circles, atovaquone ($n = 6$); red circles, atovaquone–proguanil ($n = 5$). Number and nature of the replicates as well as exact *p* values are shown in Appendix Tables S4 and S5. Source data are available online for this figure.

© The Author(s)

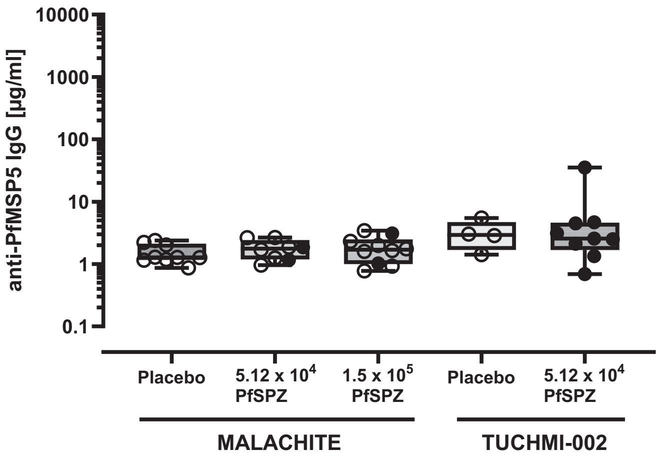

Figure EV5. Antibody reactivity against MSP5.

Reactivity of the plasma samples collected 1 day before CHMI was tested for reactivity against MSP5 in the Malachite and TüCHMI-002 studies. For quantitative analysis, reactivity was estimated in comparison to a coated human IgG standard. Boxplots display median, interquartile range (IQR), and the full range of the data. All ELISA experiments were performed in three technical replicates. No significant differences were found between the groups. Source data are available online for this figure.

 © The Author(s)
